# Supplementary material for: Transparent and attachable ionic communicators based on self-cleanable triboelectric nanogenerators
Source: Nat Commun. 2018 May 4;9:1804. doi: 10.1038/s41467-018-03954-x (PMC5935721; doi:10.1038/s41467-018-03954-x)
Supplement: Supplementary file 1 — Supplementary Information [file 41467_2018_3954_MOESM1_ESM.pdf]

Supplementary Figures

Effect of gelation time on transmittance

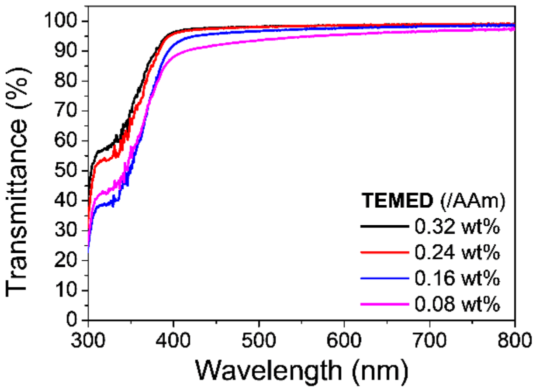

Supplementary Figure 1 | Transmittance spectra according to various weight ratio of crosslinking accelerator (TEMED) in STAICs.

N,N,N',N'—tetramethylethylenediamine (TEMED) as crosslinking accelerator affected the gelation time, resulting in transmittance difference. Transmittance increases with increasing of TEMED concentration. It was conducted with 0.8 wt% of APS.

Self-powering mechanism based on contact electrification and electrostatic induction

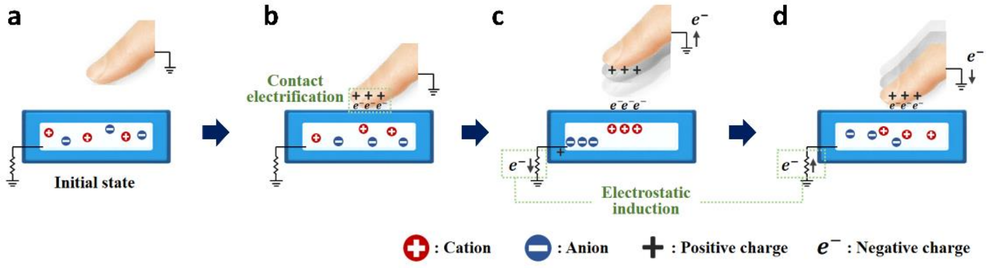

Supplementary Figure 2 | Schematic diagram showing the electrical distribution and working mechanism of the STAIC. (**a**) Original status, (**b**) contact electrification to negative triboelectric charges on PDMS, and electrostatic induction during (**c**) detaching and (**d**) attaching of the counter material.

In the initial state, there is no electric potential between the STAIC and the counter object prior to the contact between them (a). When the counter object encounters the STAIC, contact electrification occurs between them because of their relatively different electron affinities (b). When detaching, transferred charges on the contact surface attract the positive ions and repel the negative ions, which repels the electrons of inserted Pt wire due to electrical equilibrium; here, the ions in the hydrogel act as a medium. Meanwhile, the counter object obtains and electron from the ground, which enhances triboelectric output power by helping the transferred charges to repel the electrons more (c). After full separation, the attaching counter object causes the electron flow back in the reverse direction due to electrostatic induction. Meanwhile, the counter object repel electron to the ground, enhancing the output power by helping electrostatic induction of the STAIC (d).

Triboelectric performance of a STAIC contacting various materials

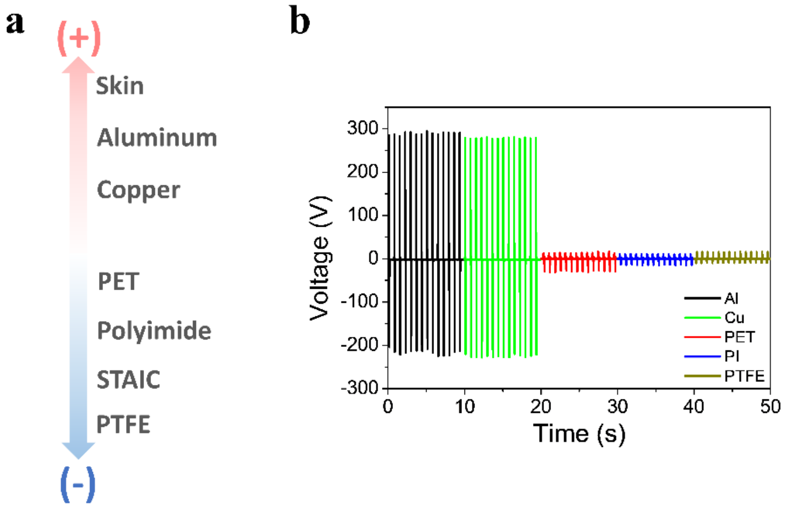

Supplementary Figure 3 | Triboelectric performances of a STAIC were investigated with various contact materials. (a) Triboelectric series. (b) Output voltages from a STAIC when touched with different materials.

We measured output voltages of a STAIC, using aluminum, copper, polyethylene terephthalate (PET), polyimide (PI) and polytetrafluoroethylene (PTFE) films as contact materials. Supplementary Figure 3a shows relative electron affinity of materials we used. Larger difference between electron affinities of contact materials led to higher triboelectric voltage outputs. When PTFE was used as a contact material, the sign of generated voltage was flipped. It can be attributed to greater electron affinity of PTFE than that of a STAIC.

The effect of time interval between consecutive contacts on the electrical output performance

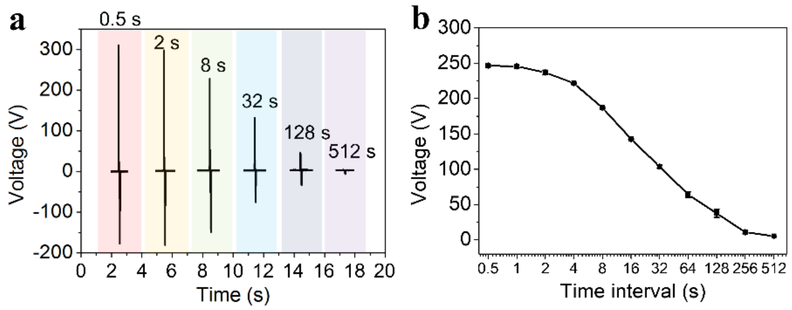

Supplementary Figure 4 | (**a**) The effect of time interval between consecutive contacts on the electrical output performances and (**b**) averaged voltages. All error bars in the figure represent s.e.m. of the data.

In order to check the effect of time interval between consecutive contacts on energy conversion, we performed experiments in which we varied duration time from 1 s to 512 s (Supplementary Fig. 4a). An increase in time interval between consecutive contacts caused output voltage to

decrease, a result which comes from charge leakage as shown in Supplementary Fig. 4b.

Anti-dehydration ability of a STAIC

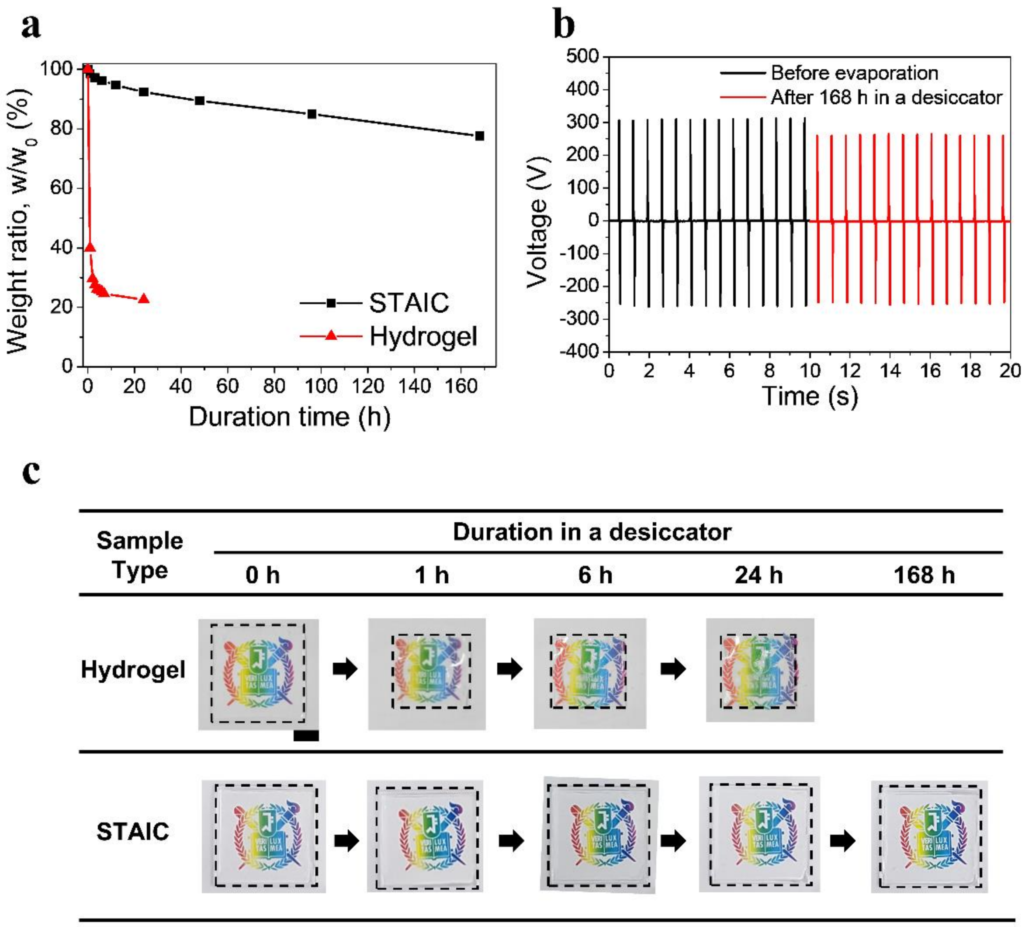

Supplementary Figure 5 | Anti-dehydration ability of a STAIC under a vacuum of 0.013 atm at room temperature. (**a**) Normalized weight ratio of a STAIC and a hydrogel according to duration time. (**b**) Triboelectric output voltages of STAICs before and after being placed under the vacuum for a week. (**c**) Photographs showing contrasted extent of shrinkages of hydrogel and STAIC during the evaporation experiments. (scale bar: 1 cm)

Triboelectric output under different strain

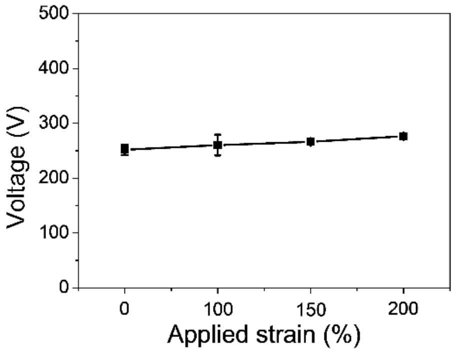

Supplementary Figure 6 | Triboelectric outputs measured under 100 %, 150 % and 200 % of applied tensile strain. All error bars in the figure represent s.e.m. of the data.

We investigated triboelectric output voltages under different strains of 100 %, 150 % and 200 %. The measured output voltages slightly increased from 252 V to 277 V when 200 % of strain was applied. The effect of applied strain can be interpreted as follows; electrostatic induction which is induced from a charged surface to an electrode can be increased when the distance between the surface and electrode becomes closer as reported by Parida et al.1 and Lai et al.2 Because a uniaxial strain reduces the thickness of the sample by the Poisson's effect, induced output voltage was increased.

Application of STAICs on touch screen

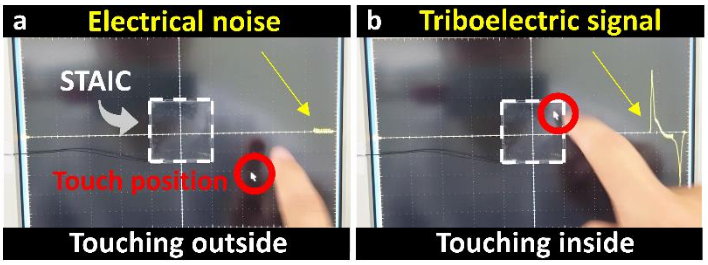

Supplementary Figure 7 | Electrical performance when touching (**a**) outside and (**b**) inside of STAIC boundary which is attached on a touch screen.

A STAIC was attached on top of a touch screen (this time, a resistive touch screen of an oscilloscope was used). A STAIC generated a voltage of 30 V when the screen was touched but did not hide the screen because the STAIC is highly transparent. Furthermore, electrical power additionally generated by the STAICs could be more beneficial if the device has limited energy supplies like a portable device.

Dynamic contact angle of HDFS treated STAICs

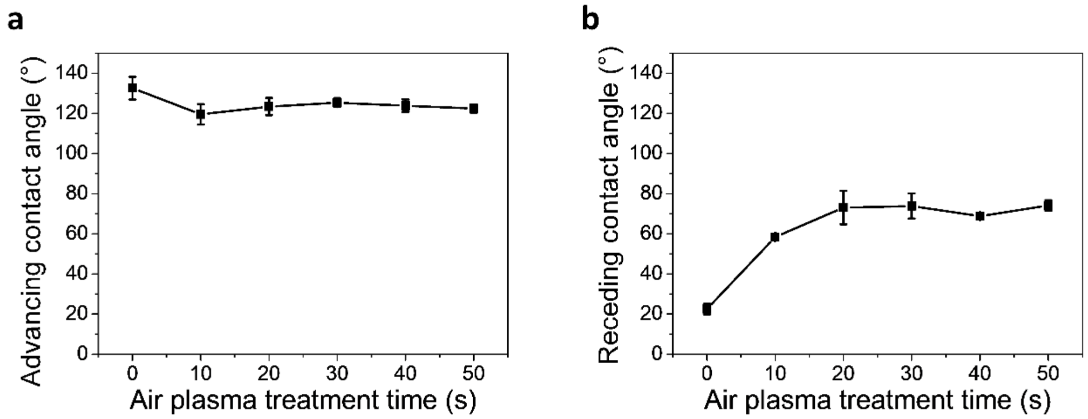

Supplementary Figure 8 | Measurement results of (**a**) advancing, (**b**) receding contact angle showing the effect of HDFS surface treatment after various plasma treatments for excited sites. All error bars in the figure represent s.e.m. of the data.

Effect of humidity on triboelectric performance

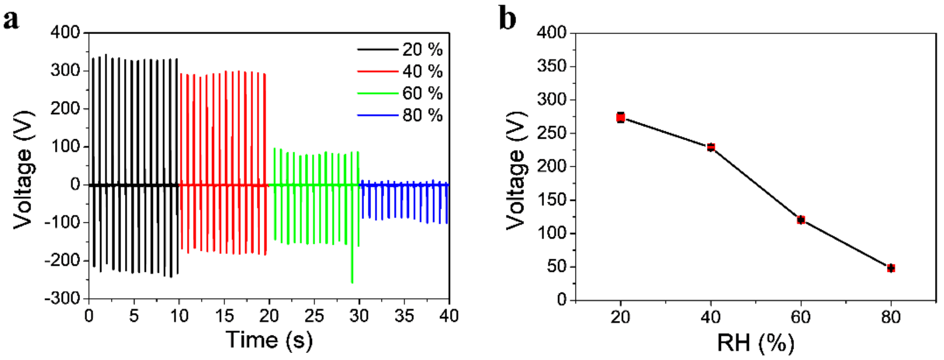

Supplementary Figure 9 | (**a**) Measured output voltages and (**b**) averaged peak to peak voltages under humid conditions. All error bars in the figure represent s.e.m. of the data.

A circuit diagram of the controller board

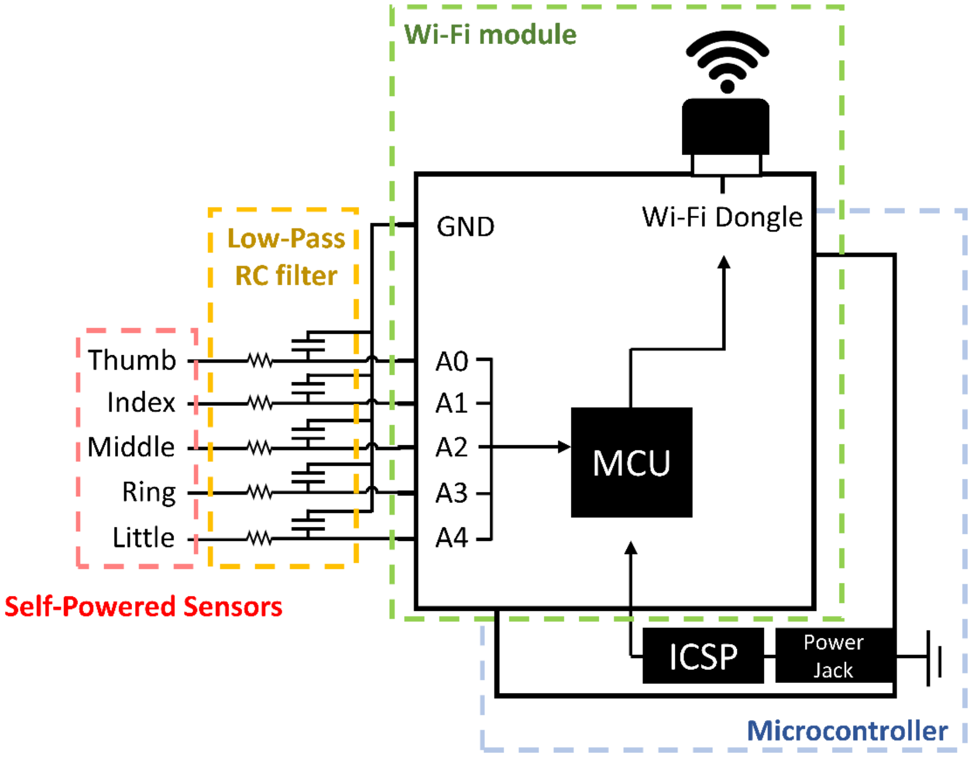

Supplementary Figure 10 | A detailed circuit diagram of the self-powered sensors and controller board.

Signal collection and processing of STAICs as human-machine interfaces

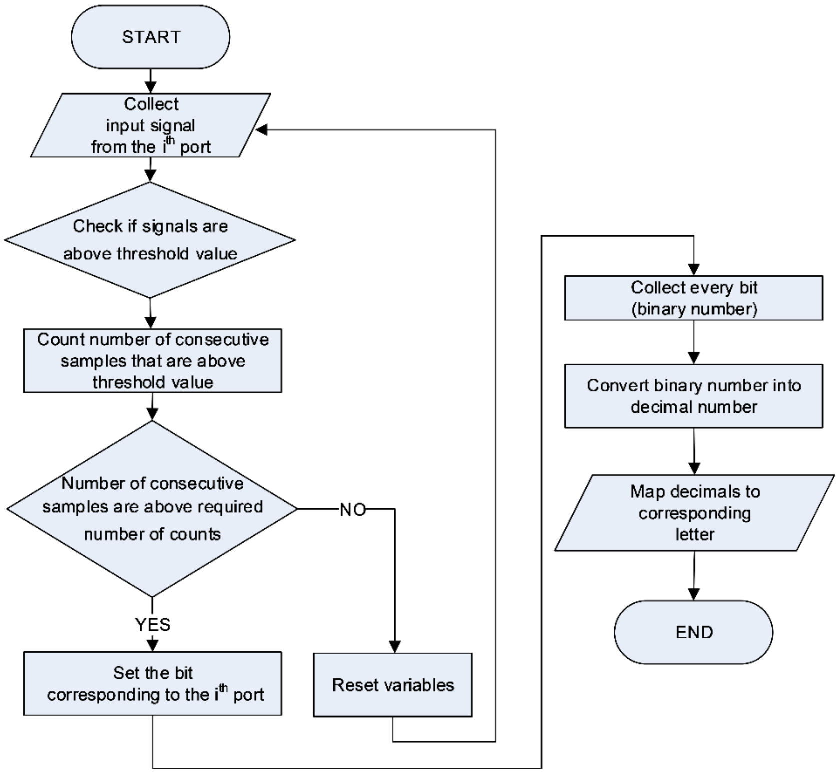

Supplementary Figure 11 | Communication processing flowchart.

A simple algorithm was developed and realized through C programming to convert a combination of finger touches into a letter and, further, words with meaning. First, the micro-controller collects the input signal, voltage, from each STAIC. The micro-controller checks whether the signals are above the threshold voltage value. Additionally, the micro-controller counts the number of consecutive samples that are above the threshold value in order to filter out the electrical signal noise. If the number of consecutive samples from an input port is above the required number of counts, the micro-controller judges that the signals are valid and sets

the bit corresponding to the input port. If the number of consecutive samples is below the boundary, the signals are determined as electrical noise, and the bit is not set. A combination of these binary bits corresponding to input ports can generate thirty-one different cases which are thirty-one different letters in our case. The micro-controller continually checks each bit corresponding to each STAIC and uses these bits to converts the binary number into the decimal number. Finally, the micro-controller maps the converted decimal number to the corresponding letter.

Supplementary references

1. Parida K, Kumar V, Jiangxin W, Bhavanasi V, Bendi R, Lee PS. Highly Transparent, Stretchable, and Self-Healing Ionic-Skin Triboelectric Nanogenerators for Energy Harvesting and Touch Applications. *Advanced Materials* **29**, (2017).

2. Lai YC*, et al.* Electric Eel-Skin-Inspired Mechanically Durable and Super-Stretchable Nanogenerator for Deformable Power Source and Fully Autonomous Conformable Electronic-Skin Applications. *Advanced Materials* **28**, 10024-10032 (2016).
