# Supplementary material for: Transparent and attachable ionic communicators based on self-cleanable triboelectric nanogenerators
Source: Nat Commun. 2018 May 4;9:1804. doi: 10.1038/s41467-018-03954-x (PMC5935721; doi:10.1038/s41467-018-03954-x)
Supplement: Supplementary file 3 — Description of Additional Supplementary Files [file 41467_2018_3954_MOESM3_ESM.pdf]

Description of Additional Supplementary Files

File Name: Supplementary Movie 1
Description: Movie showing high stretchability of a self-cleanable, transparent, and attachable ionic communicator (STAIC).

File Name: Supplementary Movie 2
Description: Movie showing self-cleaning behavior of a STAIC.

File Name: Supplementary Movie 3
Description: Movie showing self-powering behavior of a STAIC.

File Name: Supplementary Movie 4
Description: Movie demonstrating wireless real-time ionic communicators.
